# 3D Printable Dry EEG Electrodes with Coiled-Spring Prongs

**DOI:** 10.3390/s20174733

**Published:** 2020-08-21

**Authors:** Masaya Kimura, Shintaro Nakatani, Shin-Ichiro Nishida, Daiju Taketoshi, Nozomu Araki

**Affiliations:** 1 Graduate School of Sustainability Science, Tottori University, 4-101, Koyama-cho Minami, Tottori 680-8552, Japan; m20j3017k@edu.tottori-u.ac.jp (M.K.); s-nishida@tottori-u.ac.jp (S.-I.N.); 2 Advanced Mechanical and Electronic System Research Center, Faculty of Engineering, Tottori University, 4-101, Koyama-cho Minami, Tottori 680-8552, Japan; 3 Technical Department, Tottori University, 4-101, Koyama-cho Minami, Tottori 680-8552, Japan; take_mech@tottori-u.ac.jp; 4 Graduate School of Engineering, University of Hyogo, 2167 Shosha, Himeji, Hyogo 671-2201, Japan; araki@eng.u-hyogo.ac.jp

**Keywords:** electroencephalography (EEG), dry electrode, 3D printing, coiled spring, stereolithography (SLA)

## Abstract

Various dry electroencephalography (EEG) electrodes have been developed. Dry EEG electrodes need to be pressed onto the scalp; therefore, there is a tradeoff between keeping the contact impedance low and maintaining comfort. We propose an approach to solve this tradeoff through the printing of complex-shaped electrodes by using a stereolithography 3D printer. To show the feasibility of our approach, we fabricated electrodes that have flexible fingers (prongs) with springs. Although dry electrodes with flexible prongs have been proposed, a suitable spring constant has not been obtained. In this study, the spring constant of our electrodes was determined from a contact model between the electrodes and the scalp. The mechanical properties and reproductivity of the electrodes were found to be sufficient. Finally, we measured the alpha waves when a participant opened/closed his eyes by using our electrodes.

## 1. Introduction

The brain–computer interface (BCI) has been studied as a communication technology between the brain and the device [1,2,3]. Human intent has been decoded using technology such as functional magnetic resonance imaging, magnetoencephalography, functional near-infrared spectroscopy, and electroencephalography (EEG). EEG signals from the scalp using wet electrodes are widely used in communication [4,5,6], rehabilitation [7,8] due to these electrodes cost-effectiveness and high temporal resolution. However, conductive gels and glues are required to attach wet electrodes to the scalp [9], and the impedance of such gels and glues worsens over time [10], which makes it difficult to obtain stable measurements over a long period of time. Thus, wet electrodes are not suitable for daily BCI use. A dry electrode, which does not require any gel, is key for the spread of BCI technology because it reduces preparation time and enables long-term stable measurement. Therefore, various approaches for fabricating dry electrodes have been reported [11].

A well-known dry electrode, SAHARA (g.tec medical engineering GmbH, Schiedlberg, Austria), has fingers (prongs) to penetrate through the hair. A prong presses against the scalp to reduce the electrical impedance between the electrodes and skin. The impedance increases due to the decrease in contact pressure with such an electrode [12] and when it is high, the noise level of the EEG increases significantly [13]. This shows that the pressing force of the electrode should be above a certain level for stable EEG measurement. The pressing force of a dry electrode is often too invasive, and wearing them for long periods of time can cause discomfort [14]. Therefore, the greatest challenge for practical EEG measurement is to apply both an appropriate amount of pressure and maintain comfort.

There are several approaches to solve this tradeoff. The first is placing a thin needle on the tip of a prong by using microelectromechanical systems (MEMS) technology [15,16,17]. Needles are under 200 μm long, and do not reach the pain point; they penetrate only the stratum corneum (SC), which has higher impedance. By penetrating the SC with a needle, the contact impedance can be reduced even when the pressing force is low. Carbon nanotube arrays have also been used to penetrate the SC [18]. The second approach to solve this tradeoff is to add flexibility to the prongs to prevent uneven pressing force on the scalp and reduce discomfort. Soft conductive polymers [10,19], flexible carbon fine brushes [20], and prongs to include a coil [21,22] have been developed for this approach. Dispersing the pressing forces of prongs is important, not only in terms of comfort but also for EEG measurement, because the significant difference in noise levels among prongs [23] can be reduced. Mota et al. developed a small reservoir inside an electrode [24]. When a force is applied to the tip of the electrode, a small amount of conductive gel is released from the reservoir. Comparing these various approaches is not easy because they are conceptually distinct and the reported performances are not standardized [9]. A versatile and cost-effective approach for electrode fabrication is important to establish a homogenized evaluation of performance.

Due to their versatility and cost-effectiveness, 3D printers have been applied in various fields such as mechanics, electronics, and food [25] and drug delivery systems [26,27,28]. Using 3D printers is considered an easy and low-cost approach to fabricate dry electrodes. Salvo et al. fabricated a dry electrode with an array of microneedles by using a photopolymer jetting 3D printer with an XY resolution of 42 μm [29]. Fused deposition modeling (FDM) printers, which can be purchased for only a few hundred dollars, can print the shapes of EEG electrodes, and EEG can be measured by coating the printed resin with a conductive paste [30]. Velcescu et al. fabricated 3D-printed electrodes with a flexible element and obtained sufficient electrical properties [31]. However, due to the relatively coarse resolution of an FDM 3D printer, it can be difficult to produce an electrode with complex-shaped elements such as coiled springs, and the reproducibility of the mechanical properties of the electrodes have not been discussed. We can also purchase 3D printers that use stereolithography (SLA) for only a few hundred dollars. SLA 3D printers have high spatial resolution with an XY resolution of 45 μm; however, they are limited in the types of the printable resin that can be used [32]. If complex electrode shapes can be printed using an inexpensive SLA 3D printer, it will be a versatile and cost-effective approach of fabricating dry EEG electrodes. However, it is unclear whether the mechanical properties of such electrodes are acceptable for practical use.

We propose an approach involving the use of an SLA 3D printer to fabricate EEG dry electrodes with coiled-spring prongs. Specifically, we discuss a contact model between these electrodes and the scalp and obtain a suitable spring constant. We also show the process of our approach and discuss the evaluation of the mechanical and electrical characteristics of our printed electrodes. Finally, we conducted an experiment to measure EEG signals from our printed electrodes while a participant’s eyes were closed/open.

## 2. Contact Model of Electrode

**Figure 1:**
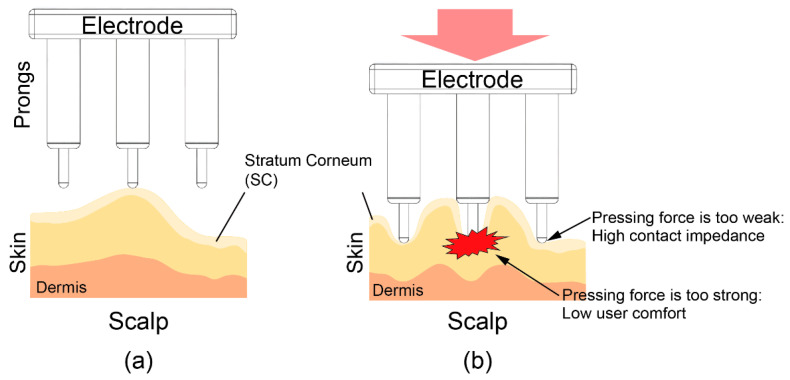
Illustration of a dry electrode and scalp. (**a**) Prongs of the dry electrode are pressed onto the scalp. (**b**) The pressing force is concentrated on a few prongs due to the uneven surface of the scalp.

To reduce the impedance between the electrode and skin, it is important that all the prongs are able to provide the necessary pressing force to the scalp. However, as shown in Figure 1, the pressing force is concentrated on a few prongs due to the uneven surface of the scalp. To reduce the impedance under these circumstances, the electrodes should be pressed hard into the scalp so that all prongs provide the necessary pressure. This strong pushing force can cause discomfort.

To solve this tradeoff, we fabricated a flexible element for each prong. This element reduces the unevenness of pressing force and reduces excessive pressing. Several dry electrodes with a similar concept have been reported; however, they are expensive and their design parameters, such as the spring constant, have not been discussed. Thus, it is difficult to compare the performances of different type electrodes. We describe the mechanical contact model between the scalp and prongs with flexible elements to obtain a suitable spring constant.

**Figure 2:**
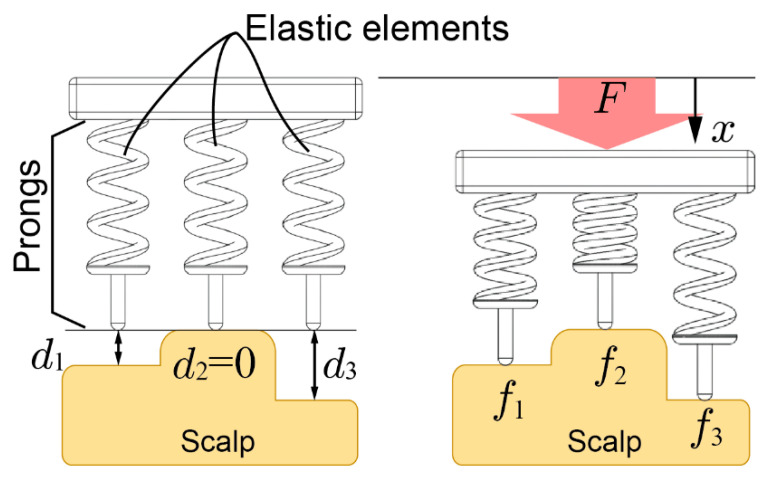
Contact model of the electrode with elastic prongs and the scalp.

Figure 2 shows 2D contact models between the scalp and an electrode. The electrode is pressed onto the scalp vertically and the top of the scalp surface is defined as the reference plane. The depth of the points that come into contact with the i-th prongs are denoted as $d_{i},$ and the deepest $d_{i}$ is denoted as $d_{\max}$. In this figure, $d_{\max}=d_{3}\geq d_{1}\geq d_{2}=0$. The contact impedance decreases by increasing pressure on the scalp [30]. However, the effect of the pressing force on decreasing the impedance is limited. Therefore, once the impedance has been sufficiently reduced, a stronger pressing force is not needed. We define this force as the minimal required force of the prong $f_{\mathrm{th}}$. When the electrode is pressed to the scalp with force F and the displacement from the reference plane is set as $x\;\left(x\geq d_{\max}\right)$, then the pressing force of the prongs are expressed as $f_{i}=k\left(x-d_{i}\right)$. To reduce impedance, all prongs should be pressed onto the scalp over the minimal required force of the prong $f_{\mathrm{im}}$ but the maximum pressing force should be minimized for comfort. Then, the allowable maximum pressing force for comfort is denoted as $f_{\mathrm{co}}$. Therefore, the following relationships should be sufficient:

$$f_{\mathrm{im}}\leq f_{i}\;\leq f_{\mathrm{co}}\left(i=1,\;2,\;\ldots \right)$$

A suitable spring constant is calculated as

$$f_{\mathrm{im}}\leq \min\left(f_{i}\right)=k\left(x-d_{\max}\right),$$

where min(⋅) is the minimum $f_{i}$. The minimum spring constant *k* can be expressed as

$$k\geq \frac{f_{\mathrm{im}}}{x-d_{\max}}.$$

Also, where max(⋅) shows maximum $f_{i}$ and maximum *k* is defined as

$$f_{\max}=kx\leq f_{\mathrm{co}}\;k\leq \frac{f_{\mathrm{co}}}{x}.$$

From these equations, the required range of *k* is expressed as

(1) $$\frac{f_{\mathrm{im}}}{x-d_{\max}}\leq k\leq \frac{f_{\mathrm{co}}}{x}.$$

The maximum x is limited by the electrode size, and $d_{\max}$ is affected by the measurement surface of the scalp. In this study, we designed the electrode by using $=2\;\mathrm{mm},\;d_{\max}=1.3\;\mathrm{mm},$ $f_{\mathrm{im}}=0.5\;N,\;\mathrm{and}\;f_{\mathrm{co}}=3\;N\;\left(\mathrm{at}\;\phi \;1.25\;\mathrm{mm}\right)$; thus, the required range of k was calculated as 0.7≤k≤1.5 N/mm. The k=1.0 N/mm.

## 3. Design and Development of 3D Printed Electrodes

We now describe the process of our approach of printing dry electrodes with coiled-spring prongs. These electrodes maintain comfort while keeping contact impedance low. The springs should be designed to have a specified *k*. We used an SLA 3D printer that includes a 2K (2560×1440 pixel) LCD display (photon-s, ANYCUBIC). This is an inexpensive 3D printer with a direct light processing (DLP) projector. The XY resolution is 45 μm, Z resolution is 25 μm, and the wavelength an LED is 405 nm. The selected parameters are not ideal, but are usable for many types of dry EEG electrodes.

### 3.1. Electrode Design

**Figure 3:**
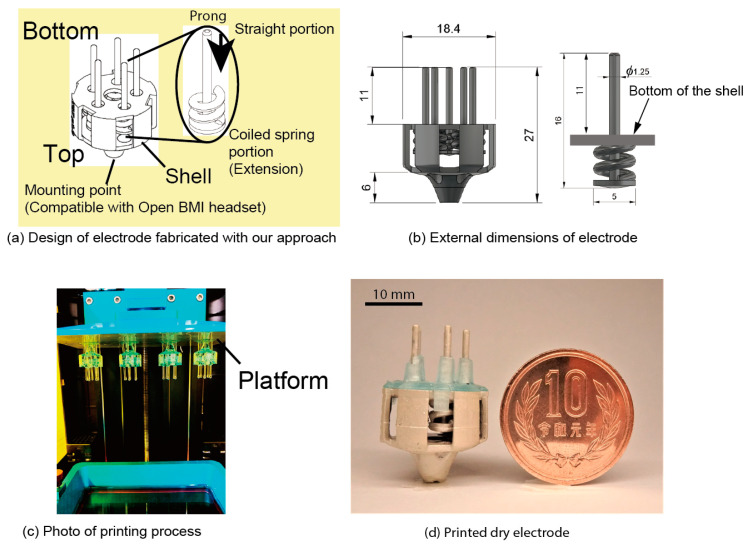
The 3D printed electrode (**a**) Design of electrode with coiled-spring prongs. (**b**) External dimensions of electrode. (**c**) Photograph of stereolithography (SLA) 3D printing process. (**d**) Electrode after being coated with conductive paste.

Figure 3a shows the design of an electrode fabricated with our approach. Dry electrodes have a shell structure, as shown in Figure 3a, and the prongs run through the shell. The coiled-spring portions of the prongs are printed from the bottom to the top of the shell. In contrast, the straight portions of the prongs are printed from the top center of the coiled spring portions toward the bottom and through the center of the coil. The specifications of the coiled spring portion (wire diameter, pitch, height, outer diameter) are determined through a mechanical characteristics test. The mounting point was designed to be compatible with the node of an EEG headset (Ultracortex Mark IV, OpenBCI). Considering the compatibility with the headset, the size of the electrode is ϕ18.4 mm in diameter and 27 mm in height, as shown in Figure 3b. The diameter of the straight portions of the prong is ϕ1.25 mm, and the length from the top of the spring to the tip is 16 mm. The coiled-spring portions are in the shell, and the length from the bottom of the shell to the tip of the prong is 11 mm. There are five prongs in the electrode.

### 3.2. 3D Printing

The process of fabricating these dry electrodes is based on that which is discussed in previous studies [30,31]. First, the electrode shape is printed using a 3D printer, then the electrode is coated with a conductive paste. The designed electrodes are printed using a strong resin (Blu, Silaya Tech, San Gabriel, CA, USA). This resin has high strength and flexibility for mechanical use. The parameters we used for printing are shown in Table 1. The top of the electrode is connected to the platform via support members, as shown in Figure 3c. With this setup, a maximum of 15 pieces can be printed and only about 7 pieces became available. After printing, electrodes are rinsed with isopropyl alcohol (IPA) (GZ901, GarageZero, Atlanta, GA, USA) and hardened with UV light (5 W, UV LED). Stiffness varies depending on the time of post-exposure, and two hours is required for stable stiffness. In this study, the exposure time was set to three hours. A conductive paste (4992N, DuPont, Wilmington, DE, USA) was used to give conductivity to the electrodes. Butyl acetate (UNNO.1123) was used for diluting, and we diluted the paste twice with butyl acetate. The diluted paste was brushed onto the resin, and natural drying was carried out for 10 min.

### 3.3. Coiled-Spring Design

**Figure 4:**
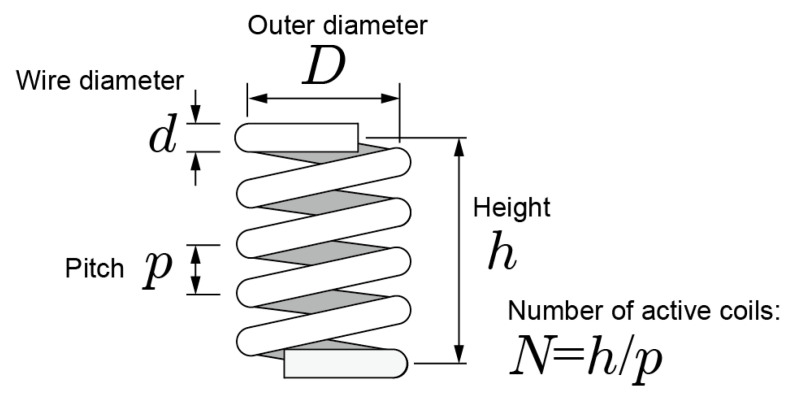
Structure of a coiled spring.

In this section, we describe the mechanical design of the coiled-spring portion of the prong. The *k* for a coiled spring is expressed as

$$k=\frac{Gd^{4}}{8N{\left(D-d\right)}^{3}}$$

where G is the modulus of transverse elasticity, which is determined by the material, N is the number of active coils, d is the wire diameter, and D is the outer diameter of the coiled spring [33]. Figure 4 shows structure of a coiled spring. The relationship between pitch p and N is shown as pN=h. Then *k* can be expressed as

(2) $$k=\frac{Gd^{4}}{8h{\left(D-d\right)}^{3}}p.$$

the spring proportions to be included in the electrodes are that the height of the spring must be h=4.2 mm, *k* must be 1.0. N/mm permissible pressing force must be 3.0 N, and D must be 5 mm. The p and d should be determined to satisfy these specifications using Equation (2). A spring test was executed to obtain the G of the resin. 

### 3.4. Mechanical Evaluation

**Figure 5:**
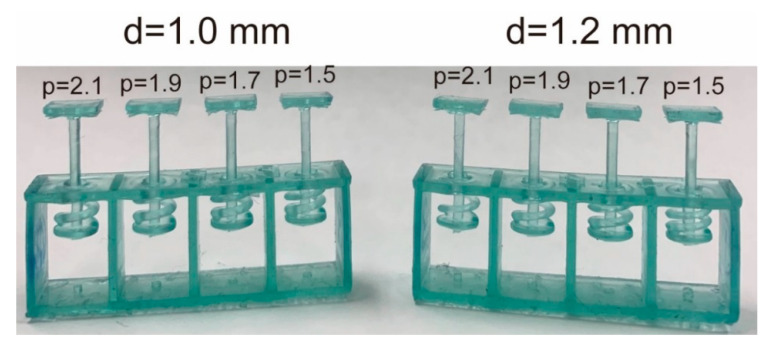
Test pieces of 3D printed coiled springs. The square plate above the prongs is the support plate used to measure the spring constant.

Figure 5 shows test springs. For the spring test, four springs were fabricated. We determined d and p to achieve the suitable *k* from the following experiments. 

Experiment 1: We first investigated *G* when we set d = 1 mm and p=1.5, 1.7, 1.9, 2.1 mm. The results indicate that *k* was too low at *d* = 1 mm. This reveals a correlation between *p* and constant and that the inclination was 0.206. The G was calculated as $4.4\times 10^{2}\;N/\mathrm{mm}$ from Equation (2). We then attempted to obtain the suitable *k* by increasing *d*. We estimated the required *d* by using Equation (2).

$$8h{\left(D-d\right)}^{3}k=pGd^{4},$$

(3) $$d^{4}+\frac{8h}{pG}k{\left(D-d\right)}^{3}=0.$$

By solving Equation (3) for k=1 N/mm and p = 1.7−2.1 mm, the required ds were 1.19−1.24 mm. We then redesigned and tested the coiled springs again with d = 1.2 mm. 

**Figure 6:**
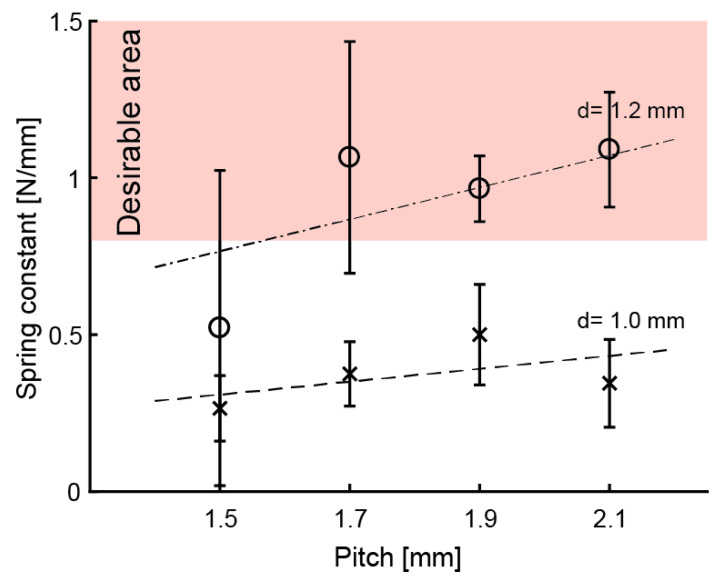
Means of spring constants. Error bar means standard deviation.

Experiment 2: Figure 6 shows the *k* for *d* = 1.2 mm and *p* = 1.5, 1.7, 1.9, and 2.1 mm. The means of *k* were around the suitable value (k=1.0 N/mm) when p = 1.7–2.1 mm. The *G* was calculated as G = 4.5 × 10^2^ N/mm, which is about the same as for *d* = 1.0 mm. Equation (2) can be used for calculating *k* from the above results. Equation (2) is also valid for springs printed with an SLA 3D printer.

### 3.5. Elasticity Evaluation of Electrode

Based on the above results, the complete electrodes were printed then checked whether the suitable *k* can be achieved. We printed four electrodes and measured the *k* of each of the five prongs. Table 2 lists the *k*s of the prongs. The mean of the *k* of each prong was 0.99 ± 0.09 N/mm. This shows that our designed coiled springs were precisely fabricated. Only two (gray) of the 20 prongs (prongs 3 and 4 of ID 4) were out of the desired range, but this electrode can be used because there is plenty of stroke.

**Figure 7:**
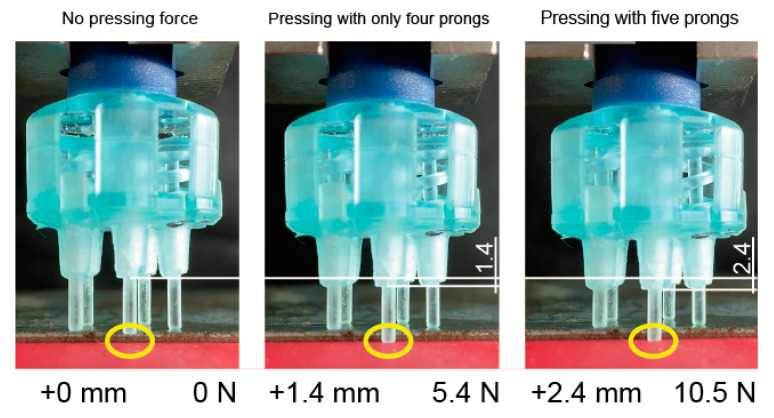
Spring constant of electrode was changed depending on the number of prongs.

Figure 7 shows an electrode pressing onto a bumpy surface. Four prongs first come into contact with the surface and only one prong remains floating (left). After pressing the electrode onto the surface with 1 mm (all prongs should be pressed over 1N in this study), the four prongs were extended and the remaining prong came into contact with the surface (middle). The pressing force was 5.4 N and the apparent *k* of the electrode was 3.9 N/mm (1.0 N/mm per prong). The pressing force then became 10.5 N when the displacement of the electrode was 2.6 mm (right). When the displacement was from + 1.4 to + 2.4 mm, the apparent *k* was calculated as (10.5–5.4 N)/(2.4–1.4 mm) = 5.1 N/mm (*k* of each prong was estimated as 5.1/5 = 1.0 N/mm). This means that the apparent *k* of the electrode was variant with the number of the contacting prongs, even though the *k* of each prong was invariant. These characteristics are valid because the electrode becomes more flexible when a few prongs come into contact with the scalp. Therefore, the other prongs can easily come into contract.

**Figure 8:**
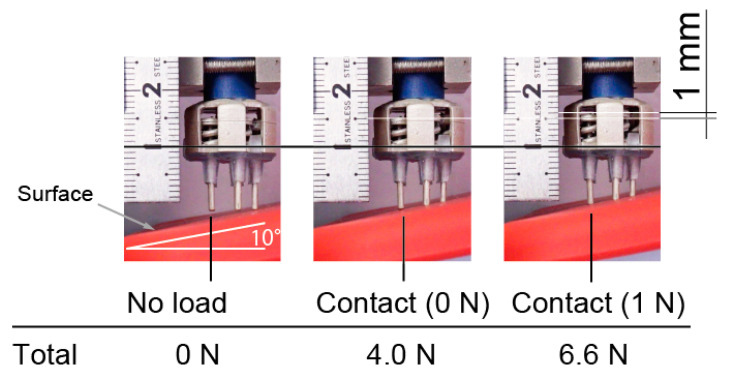
Vertical motion of prongs of electrode. All prongs were in contact with surface, and contact force was in design range.

Figure 8 shows an electrode against a slope of 10 degrees. Only the right prongs of the electrodes initially contact with the surface, then each prong come into contact with the the surface after increasing the pressing force. In this case, $d_{\max}=2.0\;\mathrm{mm}$ in Equation (1). When 1 N was applied to the leftmost prong, there was still plenty of room for the right prong’s stroke. From these results, our approach is effective.

## 4. Electrical Evaluation

**Figure 9:**
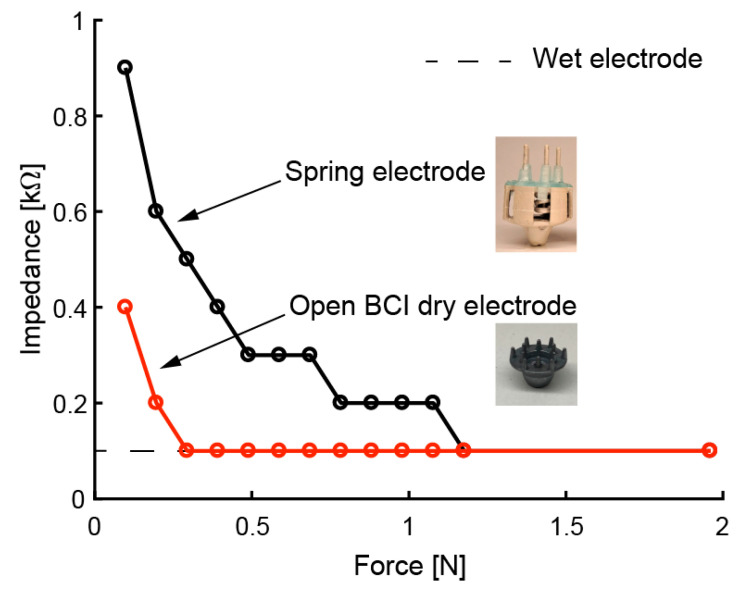
Contact impedance of electrodes when pressed against phantom head with different levels of force.

We evaluated the electrical performance of the fabricated electrodes. We expected to obtain the same performance as in previous studies [30,31] with respect to impedance, noise intensity, and drift rate due to the same approach involving coating with conductive paste. The relationship between the force on the scalp and contact impedance is affected by the elasticity of the electrode. Therefore, we measured this relationship by using a printed electrode with coiled-spring prongs, rigid dry electrodes (OpenBCI, Brooklyn, NY, USA), and wet electrodes (NE-121J, Nihon kohden, Tokyo, Japan). Based on previous studies [34,35,36], the impedance was measured on a phantom head made of gelatin and NaCl. The response of the mechanical and electrical specifications of the phantom head is said to be similar to the scalp. NaCl was mixed into water with 1% mass. The gelatin powder was mixed with hot water (60 °C) using 10% mass. In the impedance measurement of the phantom head, the electrode was pressed perpendicularly onto the phantom head. The impedance of a 30-Hz sine wave input was measured. Figure 9 shows that the impedance decreased as the electrode was pressed harder against the phantom head. In the case of the rigid dry electrode, which has twelve circular-coned prongs, 0.3 N pushing force was enough to reduce the impedance. The coiled-spring electrode with five prongs required about 1.2 N pressing force for obtaining sufficient impedance. This means that more than 0.24 N pressing force per prong is sufficient to reduce impedance for the printed electrode. These results correspond to previous results for FDM-3D-printed electrodes [30,31]. Since the $f_{\mathrm{im}}$ in Equation (1) can be set to 0.24 N, the allowable depth of the electrode is expected to be $x-f_{\mathrm{im}}/k\;=\;2\;\mathrm{mm}\;-\;\left(0.24\;N\right)/\left(1\;N/\mathrm{mm}\right)\;=\;1.76\;\mathrm{mm}$. In the future, we will be able to add a function to determine the strains of the coiled spring to indicate the pressing force on the scalp.

## 5. Functional Testing

**Figure 10:**
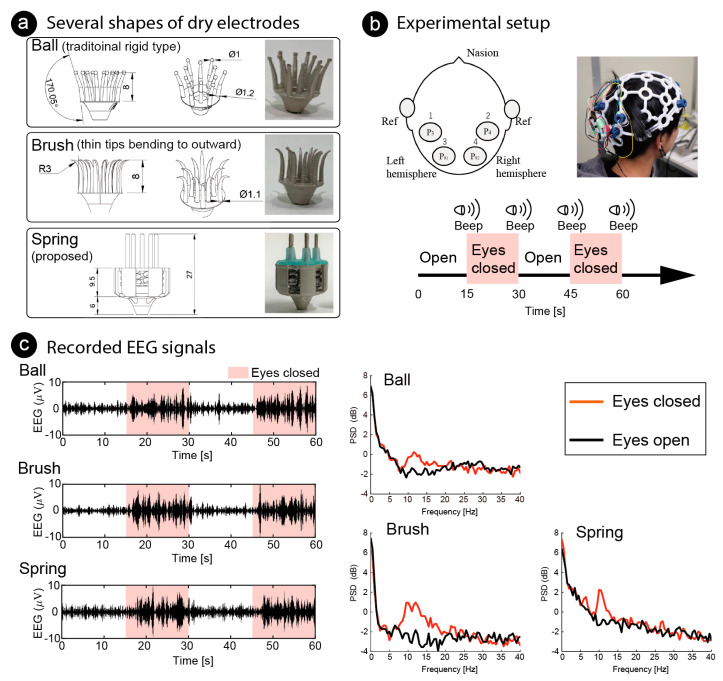
Functional testing of several shapes of dry electrodes. (**a**) Ball electrode, common shape of dry electrodes, with eleven prongs; brush electrode, designed for the bristles to bend by bending outward with pressing force, also with eleven prongs; fabricated electrode with five coiled-spring prongs. (**b**) For functional test of electrodes, electroencephalography (EEG) was measured while the participant closed/opened his eyes. Amplitudes of alpha wave (8–12 Hz) increased while eyes were closed. Dry electrodes were placed at four positions covering visual cortex and reference electrodes were placed on the earlobes. (**c**) Recorded EEG signals at P3 of PID1 are illustrated on left. Butterworth 1st-order filter (8–12 Hz) was used to emphasize alpha band. Light pink areas indicate the time the eyes remained closed, and other areas indicate the time the eyes remained open. Median of power spectrum densities (PSD) are shown on the right. Red lines show PSDs while the eyes were closed, and black lines show that while the eyes were open.

Finally, we conducted an EEG measurement for BCI by using the fabricated dry electrodes with coiled-spring prongs to measure the alpha waves. Alpha waves are known to increase after eye closure, and the phenomenon is quick (1–5 s) and reliable [37,38]. The participants were 31-year-old and 21-year-old males (two of the authors, PID1 and PID2). As shown in Figure 10a, three differently shaped electrodes were used; three fabricated electrodes with coiled-spring prongs (spring), rigid electrode with 1-mm sphere (ball), and flexible electrode with thin tips bending outward (brush). The ball electrodes were designed in the same manner as conventional dry electrodes and the elasticity of the electrodes is reliant on a spring in the holder of the headset. The brush electrodes were expected to distribute the pressure because the bristles bending by the pressing force from the headset. All electrodes were printed using an SLA 3D printer.

As shown in Figure 10b, EEG signals were recorded from the four positions located at the P3, P4, P1 and P2, based on the international 10/20 system. Reference electrodes (ear clips of Open BCI headband kit, OpenBCI, USA) were placed on the ear lobes. The electrode positions covered the visual cortex. All the electrodes were fixed to the scalp using an EEG headset (Ultracortex “Mark IV”, OpenBCI, USA). EEG signals were recorded using a wireless amplifier (Cyton Biosensing Board, OpenBCI, USA) at a sampling rate of 250 Hz. Each shape of electrode was set at the same positions on the headset. Recorded signals were applied to a common average reference (CAR) [39], which removes the common mode noise caused by a reference electrode. The EEG signals were measured at different trials for each electrode, so they would not be the same. However, we expected to see increases and decreases in alpha waves recorded from the same location in the same participant at the same time. The EEG signals were recorded while the participant closed/opened his eyes. One EEG recording trial lasted 60 s, which included two 30-s repetitions of the task: 15 s with eyes closed, followed by 15 s with eyes open. An audible beep was used as a cue. Increases in the power spectrum densities (PSD) of the alpha band were compared with one-tailed independent Welch’s t-tests. Statistical significance was set at p<0.05.

The left side of Figure 10c shows examples of the EEG signals recorded on P3 of PID1 by each electrode. The light pink areas indicate the closed-eye period, and the other areas indicate the open-eye period. A band-pass filter (Butterworth, 1st-order) of 8–12 Hz was used as the EEG signals to emphasize the amplitude of the alpha waves. Amplitude increased when the eyes were closed. Examples of frequency analysis are shown on the right of Figure 10c. To conduct frequency analysis, discrete Fourier transform (DFT) was used continuously and the median of power spectrum density (PSD) was used for comparison Window length was 2 s and overlap was 1 s. The median of PSD was used, and the PSD data around the task change was not used. 

Increases in the PSD of the alpha wave was observed for all electrodes. Table 3 shows the bands in which significant differences were found around 8–20 Hz. This indicates that a significant decrease during the eyes closed period in the alpha wave can be confirmed in all shapes of the electrodes. Participants said that the comfort of wearing the electrodes with coiled-spring prongs was clearly the highest, followed by the ball electrodes. The brush electrodes were very painful and impossible to measure for a long time. Regarding the ball electrodes, the pain was not noticeable at first, but the pain increased over time (more than 15 min of use).

## 6. Conclusions

We fabricated dry EEG electrodes with coiled-spring prongs printed using an SLA 3D printer and having sufficient reproducibility and are inexpensive. First, the contact model between the scalp and a dry electrode was discussed. We then proposed an approach of extending a prong with a coiled spring to prevent a decrease in comfort with lower impedance. Next, parameters of the mechanical elements, such as the spring constant, were estimated from the contact model. Finally, the printed dry electrodes were evaluated by conducting mechanical, electrical, and functional EEG measurements, and expected performances were achieved. The contributions of this study are as follows. (1) We defined the necessary mechanical specifications, such as a spring constant, from the contact model between the scalp and a dry electrode, (2) showed that electrodes with sufficient specifications can be fabricated using an SLA 3D printer, and (3) found that the performance of a fabricated electrode is comparable to that of a conventional electrode and that alpha waves can be detected with this electrode. The ability to fabricate dry electrodes with a reproducible flexible structure at low cost will be beneficial in various fields, such as safety driving [40,41,42], and education [43,44]. The STL file of the fabricated electrodes can be downloaded [45]. 

## Figures and Tables

**Table 1:** Printing parameters for SLA 3D printer.

Printing Parameters	Value
Layer thickness	50 μm
Normal exposure time	10 s
Bottom exposure time	75 s
Resin temperature	20–25 °C
Post-exposure time	180 min.

**Table 2:** Spring constants of prongs in printed electrodes (N/mm). Only two prongs (prongs 3 and 4 of ID 4) were out of the desired range (gray cells).

Sample	Prong 1	Prong 2	Prong 3	Prong 4	Prong 5	Mean ± S.D.
ID 1	1.07	0.90	1.15	1.17	0.72	1.00 ± 0.19
ID 2	1.42	1.08	1.25	1.39	1.22	1.27 ± 0.14
ID 3	0.90	0.73	0.82	0.77	1.27	0.90 ± 0.22
ID 4	0.78	1.03	0.61	0.68	0.73	0.76 ± 0.16

**Table 3:** The bands in which significant differences were found around 8–20 Hz (*p* *<* 0.05).

	Band with a Significant Difference (Hz)		
Participant	Ball	Brush	Spring
PID1	9.0–14.0, 15.0–17.5	8.0–19.5	9.5–13.0
PID2	10.5–12.5	10.5–12.5	9.5–10.5, 11.5–13.0
